# Association between adolescent alcohol use and cognitive function in young adulthood: A co‐twin comparison study

**DOI:** 10.1111/add.16629

**Published:** 2024-08-06

**Authors:** Megan E. Cooke, Mallory Stephenson, Sarah J. Brislin, Antti Latvala, Peter B. Barr, Maarit Piirtola, Eero Vuoksimaa, Richard J. Rose, Jaakko Kaprio, Danielle M. Dick, Jessica E. Salvatore

**Affiliations:** ^1^ Department of Psychiatry, Robert Wood Johnson Medical School Rutgers University Piscataway NJ USA; ^2^ Virginia Institute for Psychiatric and Behavioral Genetics Virginia Commonwealth University Richmond VA USA; ^3^ Institute of Criminology and Legal Policy University of Helsinki Helsinki Finland; ^4^ Department of Psychiatry and Behavioral Sciences SUNY Downstate Health Sciences University Brooklyn NY USA; ^5^ Institute for Molecular Medicine Finland FIMM University of Helsinki Helsinki Finland; ^6^ UKK Institute for Health Promotion Research Tampere Finland; ^7^ Department of Psychological and Brain Sciences Indiana University Bloomington IN USA

**Keywords:** adolescence, alcohol, cognition, intoxication, verbal ability, WAIS vocabulary, young adulthood

## Abstract

**Background and Aims:**

Studies on adolescent alcohol use and cognition are often unable to separate the potential causal effects of alcohol use on cognition from shared etiological influences, including genetic influences or other substance use comorbidities also known to be associated with cognition, such as nicotine use. The present study aimed to fill this gap and clarify the relationship between adolescent alcohol use and young adult cognition by accounting for both measured and unmeasured confounders.

**Design:**

A random effects model accounting for nesting in families was used to control for measured confounders. Next, co‐twin comparisons were conducted within the full sample and in monozygotic twin pairs (MZ) to control for unmeasured genetic and environmental confounders shared by co‐twins.

**Participants/Setting:**

Participants were 812 individuals (58.6% female, 361 complete pairs, 146 MZ pairs) from the longitudinal FinnTwin12 study in Finland.

**Measurements:**

Adolescent alcohol use was indexed with measures of frequency of use and intoxication averaged across ages 14 and 17. Cognitive outcomes were measured at average age 22 and included Trail Making Test, California Stroop test, Wechsler Adult Intelligence subtests (Vocabulary, Block Design, Digit Symbol), Digit Span subtest of Wechsler Memory Scale, Mental Rotation Test and Object Location Memory test. Covariates included sex, parental education, general cognitive ability, current alcohol use and nicotine use.

**Findings:**

Greater frequency of alcohol use and frequency of intoxication across adolescence was associated with decreased vocabulary scores in the co‐twin control [freq: stnd beta = −0.12, 95% confidence interval (CI) = −0.234, −0.013] and MZ only co‐twin control models (freq: stnd beta = −0.305, 95% CI = –0.523, −0.087; intox: stnd beta = −0.301, 95% CI = ‐0.528, −0.074).

**Conclusions:**

In Finland, there appears to be little evidence that adolescent alcohol use causes cognitive deficits in young adulthood, except modest evidence for association of higher adolescent alcohol use with lower young adult vocabulary scores.

## INTRODUCTION

Alcohol is widely used among adolescents and young adults. The neurotoxic properties of alcohol and the continuing neuromaturation of the brain during adolescence and young adulthood [1, 2, 3] set the stage for cognitive consequences in the form of both acute and sustained cognitive impairments [4, 5, 6, 7, 8]. However, previous studies demonstrating cognitive deficits from alcohol use rarely separate the potential causal effects of alcohol use on cognition from shared etiological influences, including genetic influences, or other substance use comorbidities also known to be associated with cognition, such as nicotine use [9, 10, 11]. Therefore, testing the effect of adolescent alcohol use on cognition, above and beyond the effects of shared genetic or environmental influences or comorbid nicotine use is important for clarifying whether adolescent alcohol use is likely to be a causal, modifiable risk factor for cognitive deficits in young adulthood.

Longitudinal research has assisted in parsing, which cognitive differences predate alcohol use, and therefore, may predispose the individual to subsequent alcohol use and cognitive differences that may be the result of or caused by early and/or heavy alcohol use. For example, neurocognitive and personality factors related to inhibition and impulsivity have been consistently shown to predate alcohol use [12, 13, 14]. Prospective studies examining adolescent alcohol use and cognitive functioning 6 to 10 years later found higher levels of alcohol use were negatively associated with verbal learning, memory and attention [15, 16, 17] even after controlling for baseline cognitive performance.

Importantly, however, none of these studies included potential genetic confounders in the relationship between adolescent alcohol use and cognitive outcomes. Accounting for the potential confounding effects of genetics is essential, because both alcohol use and cognitive ability are influenced, in part, by genetic factors [18, 19, 20]. Additionally, there is evidence of shared genetic factors that contribute to both alcohol use disorder and lower cognitive ability [21, 22].

Another potential confounding factor in the relationship between alcohol use and cognitive ability is nicotine use. Comorbid alcohol and cigarette use is particularly prevalent, especially among heavy alcohol users [23, 24, 25]. The acute effects of smoking/nicotine use improve some areas of cognition, specifically attention and memory [26, 27]. Yet, similar to long‐term heavy alcohol use, persistent smoking is associated with cognitive deficits [9, 10, 11, 28], potentially because of selection effects [29]. The high comorbidity of alcohol and nicotine use combined with the unique associations between nicotine use and cognitive outcomes underlines the importance of considering nicotine use when examining the relationship between adolescent alcohol use and cognition.

The goal of the current study is to further understand the potentially causal relationship between alcohol use in adolescence and young adult cognitive performance. Using data from the FinnTwin12 cohort study [30], an ongoing longitudinal study of Finnish twins recruited into the study at age 12, we sought to first quantify the association between adolescent alcohol use and cognition in young adulthood at the individual level and next determine the extent to which these relationships are robust through successively stricter control for potential confounders at the level of (1) known measurable confounders (current daily smoking, education level, parental education level); and (2) latent familial factors (i.e. genetic and environmental factors that co‐twins share). We hypothesized that greater adolescent alcohol use, both frequency of consumption and frequency of intoxication, would be significantly associated with poorer cognitive outcomes despite inclusion of the measured confounders described above. We further hypothesized that the magnitude of the relationship between adolescent alcohol use and cognitive outcomes would be attenuated in the co‐twin analyses indicating some familial confounding (both genetic and environmental).

## METHODS

### Sample

Participants came from FinnTwin12 (FT12), a population‐based study of Finnish twins born 1983 to 1987 and identified through Finland's Central Population Registry [30]. For the current study, our analytic sample included a subset of intensively studied individuals embedded within the larger epidemiological sample. These individuals were mostly randomly selected, but enriched for twins with an elevated familial risk for alcohol use disorders [30, 31]. In addition to completing surveys administered to the epidemiological sample at ages 12, 14 and 17, intensive sample participants were invited to participate in interviews and in‐person testing as adolescents (mean age, 14, *n* = 1854) and as young adults (mean age, 22, *n* = 1347). In total, 812 individuals completed an in‐person cognitive functioning battery at age 22. Twin pair zygosity was initially assessed by parents using a standardized set of questions on similarity [32] and later confirmed by genetic testing. The analytic sample included the following number of complete twin pairs: 99 monozygotic female twin pairs (MZF), 47 monozygotic male twin pairs (MZM), 71 dizygotic female twin pairs (DZF), 50 dizygotic male twin pairs (DZM) and 94 opposite sex dizygotic twin pairs (DZO).

### Measures

#### Adolescent Alcohol Use

Frequency of alcohol use and frequency of intoxication were measured at ages 14 and 17 from self‐report questionnaires. Consistent with previous reports using this sample [33, 34], categorical responses were recoded to the number of days per month a participant drank alcohol (frequency of alcohol use) and the number of a days per month a participant got ‘really drunk’ (frequency of intoxication) at each age. Composite measures of frequency of alcohol use and frequency of intoxication were created by averaging the age 14 and age 17 timepoints for each variable, separately.

#### Young Adult Cognitive Measures

During the age 22 assessment, participants completed neuropsychological tests through traditional in‐person one on one assessment and group assessment. Table 1 shows the cognitive measures that were the main outcomes of interest. Further information about each task is reported in the Supplemental Methods and in Vuoksimaa et al. [42] We note that the object location memory task was added to the cognitive battery midway through data collection and is, therefore, only available on a subset of the sample (*n =* 339).

**TABLE 1:** Neuropsychological outcomes.

Outcome	Test	Description	Construct	Reference
Trails A	Trail Making test	Connect numbered targets in order	Processing speed	Tombaugh [35]
Trails B	Trail Making test	Connect numbered and lettered targets in order alternating numbers and letters	Set‐shifting	Tombaugh [35]
Classic Stroop	Stroop test	Read aloud the color of the ink not the name of the color	Inhibition	Stroop [36]
California Stroop	Stroop test	Same as above unless the word is in a rectangle then read aloud the name of the color	Set‐shifting	Comalli *et al*. [37]
Vocabulary	Wechsler Adult Intelligence Scale (WAIS‐R)	Correctly define words presented	Verbal comprehension	Wechsler [38]
Block design	Wechsler Adult Intelligence Scale (WAIS‐R)	Correctly arrange blocks to make the pattern depicted	Perceptual organization	Wechsler [38]
Digit symbol	Wechsler Adult Intelligence Scale (WAIS III)	Match symbols to numbers to transcribe a code	Processing speed	Wechsler [38]
Digit span forward	Wechsler Memory Scale‐R	Repeat numbers in the order presented	Working memory	Wechsler [39]
Digit span backward	Wechsler Memory Scale‐R	Repeat numbers in the reverse order presented	Working memory	Wechsler [39]
Mental rotation test	Mental rotation test	Mentally rotate two‐dimensional drawings of three‐dimensional objects	Spatial processing	Peters *et al*. [40]
Object location memory	Object location memory	Identify whether an object is in a new location	Incidental memory	Silverman and Eals [41]

*Note*: The Trails B and Stroop outcomes were also analyzed as scores adjusted for performance on Trails A and the simpler Stroop tasks. The results were not substantively different from the unadjusted scores presented here. The object location memory test was only available on a subset of participants (*n =* 339) because of the task being added to the cognitive battery midway through data collection.

#### Covariates

We adjusted for a series of covariates and potential confounders previously shown to be associated with cognitive outcomes including sex, smoking status, concurrent alcohol use, parent's highest level of educational attainment and either prior cognitive performance or age 12 grades depending on availability of data [7, 8, 9, 10, 43].

Participant sex was identified from population registry data. Lifetime smoking status was measured at age 22 by asking participants about their current smoking habits. Categorical responses were recoded to fit into one of four categories: never smoker, former smoker, occasional smoker and current daily smoker.

Alcohol use at age 22 was measured by asking participants how many servings of beer, wine or spirits they typically drink on each day of the week. These drink counts were then summed across beverage type and days of the week to create a measure of drinks per week at age 22.

Parental education was included as a covariate to account for both family socio‐economic status (SES) as well as parental cognitive ability. Mothers and fathers were each asked two questions about their education as part of the parent survey administered when the twins were age 12. Consistent with a previous report [6], these two questions were combined into one parental education variable represented by the following categories: compulsory education only, vocational secondary education, academic secondary education and tertiary education. The highest level of education reached by either parent was used for subsequent analyses.

To account for pre‐existing individual cognitive differences, we included age 14 cognitive data where appropriate (for Trail Making Test and Stroop outcomes, see Supplemental Methods for additional descriptions) and age 12 academic achievement for tasks that had not been administered at age 14. The twins' academic achievement was reported by their teacher at age 12. Teachers reported each twin's grade point average using the Finnish GPA system, which ranges from 4 to 10. Their responses were then categorized to ‘below 6.0’, ‘between 6.0 and 6.9’, ‘between 7.0 and 7.9’, ‘between 8.0 and 8.9’ and ‘better than 9.0’. For twins who did not have a teacher reported GPA at age 12, their GPA was imputed using other teacher reported metrics of school performance [6, 44].

#### Analytic Plan

Our research questions and analytic plan were pre‐registered (osf.io/zje4w). For each cognitive outcome, we ran a set of analyses quantifying the relationship between adolescent frequency of alcohol use or intoxication with successively stricter control for potential confounders. First, we ran correlations to examine the unadjusted associations between our adolescent alcohol predictors and the young adult cognitive outcomes. Next, we ran a random effects model, using the ‘random’ option in the model argument of the plm package [45], which assessed the association of adolescent alcohol use (frequency of alcohol use and frequency of intoxication, separately) with cognitive outcomes while accounting for the nesting of individual twins within their families and adjusting for biological sex, age 22 drinks per week, age 22 smoking status, parental education and cognitive ability (cognitive outcomes at age 14 or GPA at age 12) as covariates.

Next, the within family effects were estimated in two stages using a co‐twin control model specified through the ‘within’ option of the model argument in plm. The overall within family estimate of the co‐twin control model (sometimes referred to as twin or family fixed‐effects) represents the association between the alcohol use variable and cognitive outcome after controlling for all environmental and partial genetic factors that vary between families. This model also included biological sex, age 22 drinks per week, age 22 smoking status, parental education and a measure of general cognitive ability as covariates. Finally, we ran a co‐twin control model in only the monozygotic (MZ) twins with the same covariates as the previous co‐twin control model. This is the most conservative test of the nature of the association between adolescent alcohol use and young adult cognitive outcomes owing to the reduced sample size and through complete control of potentially confounding genetic factors because of MZ twins sharing all their genetic variation at the genome sequence level. A false discovery rate (FDR) correction was performed within each analytic level (correlation, individual level, co‐twin, MZ only) to adjust for multiple testing using the p.adjust function.

*Post hoc* sensitivity analyses were conducted to determine whether our results varied because of the inclusion of age 22 alcohol use or the measurement of adolescent alcohol use. To examine this, we reran the individual level models without the age 22 drinks per week covariate for each cognitive outcome. In a second set of sensitivity analyses, we examined whether an alternative operationalization of the adolescent alcohol use measures affected the results. For these analyses, we used maximum frequency of alcohol use or intoxication as the predictor, rather than average frequency.

## RESULTS

Descriptives for the analytic sample are summarized in Table 2 (descriptives by zygosity are shown in Table S1). With respect to adolescent alcohol use, 104 twins (12.8%) reported not drinking alcohol at either the age 14 or age 17 surveys and 175 twins (21.6%) reported not drinking to intoxication at either the age 14 or age 17 surveys. Adolescent alcohol frequency and adolescent intoxication were significantly correlated (r = 0.68, CI = 0.64–0.72). With respect to young adult alcohol use, 30 twins (3.7%) reported not drinking during any of the timepoints reported here (ages 14, 17 and 22).

**TABLE 2:** Sample Descriptives.

	Male	Female	Total
	*n* = 336	*n* = 476	*n* = 812
MZ	109 (32.44%)	206 (43.28%)	315 (38.79%)
DZ	116 (34.52%)	149 (31.30%)	265 (32.64%)
DZO	110 (32.74%)	121 (25.42%)	231 (28.45%)
Unknown zygosity	1 (0.30%)	0 (0.00%)	1 (0.12%)
Age 14 alcohol use frequency	0.40 (0.87)	0.44 (0.81)	0.42 (0.83)
Age 14 intoxication frequency	0.17 (0.42)	0.26 (0.65)	0.22 (0.57)
Age 17 alcohol use frequency^a^	2.14 (2.34)	1.99 (2.10)	2.05 (2.20)
Age 17 intoxication frequency	0.84 (1.19)	0.82 (1.19)	0.83 (1.19)
Age 22 cognitive outcomes			
Trails A time	28.52 (9.60)	29.82 (9.45)	29.28 (9.53)
Trails B time	63.95 (25.92)	61.60 (21.72)	62.58 (23.56)
Classic Stroop*	35.08 (11.07)	32.36 (8.18)	33.49 (9.57)
California Stroop*	42.54 (10.82)	38.39 (9.90)	40.12 (10.49)
Vocabulary*	18.3 (4.98)	19.4 (4.71)	18.97 (4.85)
Block design	39.0 (7.96)	37.8 (8.27)	38.28 (8.16)
Digit symbol*	77.9 (14.9)	84.4 (14.3)	81.70 (14.89)
Digit span ‐ forward	7.24 (1.92)	7.20 (1.78)	7.22 (1.84)
Digit span ‐ backward	6.59 (1.73)	6.52 (1.59)	6.55 (1.65)
Mental rotation test score* ^,^ ^a^	13.15 (4.86)	9.33 (4.20)	10.92 (4.86)
Object location memory*	13.28 (6.14)	15.22 (5.29)	14.36 (5.76)
Highest parental education level^a^			
Compulsory education			56 (6.89%)
Vocational secondary education			265 (32.64%)
Academic secondary education			273 (33.62%)
Tertiary education			211 (25.99%)
Age 22 drinks per week*	11.18 (10.40)	5.70 (4.89)	7.97 (8.12)
Age 22 smoking status			
Never smoker	40 (11.9%)	87 (18.28%)	127 (15.64%)
Former smoker	156 (46.43%)	215 (45.17%)	371 (45.69%)
Occasional smoker	40 (11.9%)	52 (10.92%)	92 (11.33%)
Daily smoker	98 (29.17%)	119 (25.0%)	217 (26.72%)
Age 12 teacher's reported GPA*			
Below 6	0 (0%)	0 (0%)	0 (0%)
Between 6.0–6.9	16 (4.76%)	9 (1.89%)	25 (3.08%)
Between 7.0–7.9	127 (37.80%)	139 (29.20%)	266 (32.76%)
Between 8.0–8.9	158 (47.02%)	278 (58.40%)	436 (53.69%)
Over 9.0	9 (2.68%)	28 (5.88%)	37 (4.56%)

*Note*: Means and SD are reported above for continuous variables while *n* and % of the sample are reported for categorical variables.

Abbreviations, DZ, dizygotic; DZO, opposite sex dizygotic twins; GPA, grade point average; MZ, monozygotic.

* A significant difference between males and females at *P* < 0.05,

^a^ A significant difference between MZ and DZ twins at *P* < 0.05.

In what follows, we present the associations between young adulthood cognitive outcomes and adolescent alcohol use or intoxication frequency separately, moving from unadjusted associations at the population level (zero order correlations) to a model adjusted for covariates to within pairs (all pairs and MZ co‐twin control). This permits examination of the associations between the adolescent alcohol measures and young adult cognitive outcomes, accounting for increasing levels of control for measured and unmeasured (genetic and environmental) confounding.

### Frequency of Alcohol Use

**FIGURE 1:**
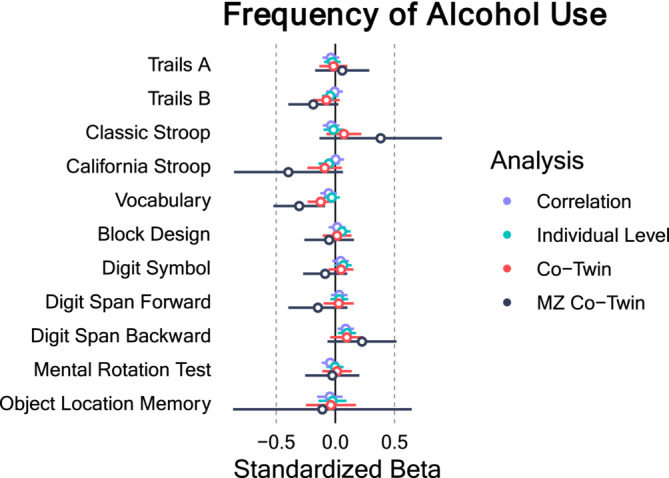
Association between frequency of alcohol use and cognitive outcomes. Note: Error bars represent 95% CI. Filled in circles represent false discovery rate corrected *P* < 0.05.

Figure 1 shows the results of the standardized estimates of the relationship between frequency of alcohol use and each cognitive outcome (full model results are shown in Tables S2–S12, FDR corrections are shown in Table S13). Most of the zero order correlations were small (|r| = 0.004–0.087) with the largest being between frequency of use and backward digit span (r = 0.087, 95% CI = 0.018–0.157, FDR corrected *P* = 0.157). The individual level effects from the random effects model did not substantively differ from the zero order correlations. The within family effect of the co‐twin control models were very similar to the estimates from the zero order correlations and between family effects, with the exception of the vocabulary scale, where the within family effects were stronger [standardized (std) β = −0.124, 95% CI = −0.234 to −0.013, FDR corrected *P* = 0.322] than the correlation or individual level estimates (r = −0.056, std β = −0.030). The within family effects of the MZ only co‐twin control model for vocabulary scores were even stronger in this model than the previous models (std β = −0.305, 95% CI = −0.523 to −0.087, FDR corrected *P* = 0.079), indicating that more frequent adolescent alcohol use was associated with lower vocabulary scores after controlling for genetic and environmental factors that co‐twins share.

### Frequency of Intoxication

**FIGURE 2:**
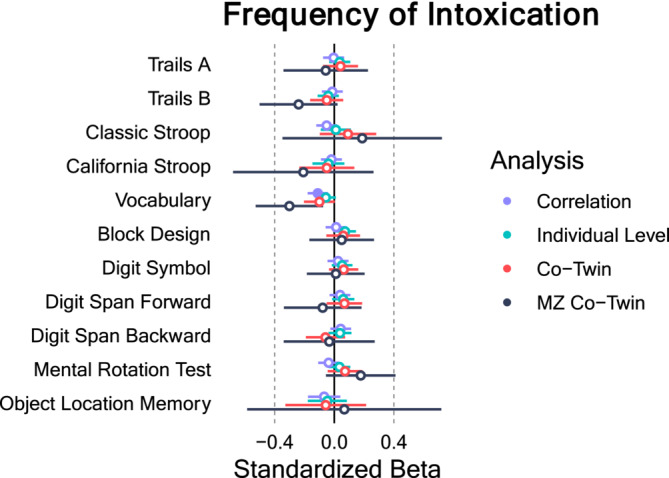
Association between frequency of intoxication and cognitive outcomes. Note: Error bars represent 95% CI. Filled in circles represent false discovery rate corrected *P* < 0.05.

Figure 2 shows the estimates of the zero order correlations, individual level analyses, co‐twin control models and MZ only co‐twin control models in purple, teal, red and black, respectively (full model results are shown in Tables S2–S12, FDR corrections are shown in Table S14). Most of the zero order correlations were small (|r| = 0.004–0.111), with the largest being vocabulary score (r = −0.111, 95% CI = –0.179 to −0.042, FDR corrected *P* = 0.021). In the adjusted individual analyses, the estimate of the association between frequency of intoxication and vocabulary score was attenuated (std β = −0.057 95% CI = −0.124 to 0.011, FDR corrected *P* = 0.375) after controlling for sex, age 22 drinks per week, age 22 smoking status, parental education and teachers' report of age 12 GPA. The estimates from the individual level models remained generally unchanged from the zero order correlations for all other cognitive outcomes. Regarding the co‐twin control models, the majority of the within family estimates were similar to the estimates from the zero order correlations and individual level estimates. The estimates from the MZ only co‐twin control model (shown in black), which represent the strictest control of the association between frequency of intoxication and each cognitive outcome, produced a stronger negative effect for vocabulary scores than the previous models (std β = −0.301, 95% CI = −0.528 to −0.074, FDR corrected *P* = 0.115).

### Sensitivity Analyses

The exclusion of the age 22 drinks per week covariate did not substantively change the results or interpretation of the findings described above (Figure S1). Similarly, sensitivity analyses substituting maximum adolescent frequency of alcohol use or intoxication as the predictor of interest did not result in substantive changes from the original individual level results (Figure S2).

### Moderation by Sex

At the request of a reviewer, we examined biological sex as a moderator of effects; however, we did not find strong evidence of interactions (Table S15).

### *Post hoc* Power Analyses

We used the pwr package in R to determine that at a sample size of 812 twins we had 80% power to detect zero‐order correlations of 0.098 or larger before FDR correction. We used the simr package to determine we had 80% power to detect estimates from a random effects model of 0.1 or larger before FDR correction in a sample of 812 twins.

## DISCUSSION

In the current study, we sought to quantify the associations between adolescent alcohol use and cognitive outcomes in young adulthood while controlling for measured and latent familial confounders. To accomplish this, we used longitudinal data and the twin design of the FinnTwin12 study, which is useful in controlling for both measured and unmeasured potential confounders. Overall, we found the greatest effect between both greater frequency of alcohol use and greater frequency of intoxication in adolescence with lower vocabulary scores, with effect sizes progressively increasing across levels of analysis. However, these associations did not survive an FDR correction. There was little to no statistically significant relationship with adolescent alcohol use for the other dimensions of cognitive functioning.

The relationship between problem alcohol use and verbal ability has been previously documented [4, 15]. In a cross‐sectional analysis, adolescents in treatment for alcohol use disorder performed worse on the the Wechsler Intelligence Scale for Children‐Revised vocabulary test than controls matched for age, sex, SES, education and family history of substance use disorders [4]. Particularly relevant to the current findings, a longitudinal study starting in adolescence and following participants for 10 years into young adulthood found that greater cumulative alcohol use throughout the follow‐up period was associated with lower verbal learning and memory scores in adulthood [15]. However, these studies did not account for the potential confounding of shared genetic factors between verbal ability and alcohol use [21]. Our findings in the MZ only sample as well as an increase in the magnitude of effect over the estimates from the individual level and co‐twin analyses are consistent with prior literature, which suggests a causal effect of adolescent alcohol use on verbal ability (measured by Wechsler Adult Intelligence Scale vocabulary scores). The fact that this association was observed for both frequency of use and frequency of intoxication suggests that a high frequency of alcohol use in adolescence may be just as harmful as a high quantity. Frequency of use and intoxication are also moderately correlated in adolescence (r = 0.68 in our sample).

Besides verbal ability, we did not observe strong consistent evidence of associations between adolescent alcohol use and any other young adult cognitive outcome across analyses with progressively stricter levels of covariate control. In fact, many of the associations between cognitive outcomes and adolescent alcohol use hovered around zero despite *post hoc* power analyses indicating that we had 80% power to detect correlations of 0.098 or larger and estimates in the individual level model of 0.1 or larger in the full sample. This lack of associations is especially surprising for the Block Design, Digit Span, Digit Symbol, Trail Making and Stroop tests as previous prospective studies of adolescents have noted deficits in these tests associated with long‐term alcohol use [15, 16, 17]. Considering the findings of the current study, these previous associations may have been because of familial confounding either from genetic or environmental factors meaning the causal effect sizes may be small. However, replication of the current study's findings is needed to build confidence in the non‐causal relationship between adolescent alcohol use and these areas of young adult cognition.

Our results were bolstered by several strengths of the study such as leveraging the relatedness of twins to conduct quasi‐causal analyses, the prospective data collection across 10 years and the relatively large sample size for neuropsychiatric data. Despite these strengths, the results of this study should be interpreted in the context of several limitations. First, the MZ only analyses provide both the strongest level of control and are conducted in the smallest sample sizes making modest effects difficult to detect. Therefore, as with any null result, null findings in the MZ only analyses should not be interpreted as evidence for no effect, but rather that any association is of very modest magnitude [46]. Although the longitudinal prospective nature of the dataset is a clear strength, the gaps between when the data was collected are quite large (age 14 to age 17 = 3 years, age 17 to age 22 = 5 years). It is possible that with this method of data collection heavier (or lighter) drinking patterns that began and ended between the data collection time points could be missed. Finally, the co‐twin control method accounts for potential confounders that are shared between the twins. It does not account for potential confounders that may differ between twins. Although we included measures of potentially confounding factors including current alcohol use and lifetime smoking status and adolescent cognitive ability in each model, additional factors such as other substance use or differences in parenting that may affect both alcohol use and cognition and can vary across twins may still influence the results of these analyses.

Overall, our findings did not support a direct causal effect of both frequency of alcohol use and frequency of intoxication during adolescence on cognitive measures in young adulthood despite the previously demonstrated neurotoxic effects of alcohol [47, 48, 49, 50, 51]. The absence of robust associations in our sample illustrates the complexity of translating effects seen in neuroanatomy or neurological functioning to functional phenotypic deficits; further underscoring the importance of accounting for potentially confounding genetic and shared environmental influences as well as the need for replication.

## AUTHOR CONTRIBUTIONS

**Megan E. Cooke:** Conceptualization (lead); formal analysis (lead); visualization (lead); writing—original draft (lead); writing—review and editing (equal). **Mallory Stephenson:** Conceptualization (equal); formal analysis (supporting); writing—review and editing (equal). **Sarah J. Brislin:** Conceptualization (equal); writing—review and editing (equal). **Antti Latvala:** Conceptualization (equal); writing—review and editing (equal). **Peter B. Barr:** Conceptualization (equal); writing—review and editing (equal). **Maarit Piirtola:** Writing—review and editing (equal). **Eero Vuoksimaa:** Conceptualization (equal); writing—review and editing (equal). **Richard J. Rose:** Conceptualization (equal); writing—review and editing (equal). **Jaakko Kaprio:** Conceptualization (equal); funding acquisition (equal); supervision (equal); writing—review and editing (equal). **Danielle M. Dick:** Conceptualization (equal); funding acquisition (equal); supervision (equal); writing—review and editing (equal). **Jessica E. Salvatore:** Conceptualization (equal); funding acquisition (equal); supervision (lead); writing—review and editing (equal).

## DECLARATION OF INTERESTS

None.

## Supporting information


**Table S1.** Sample Descriptives by Zygosity. Means and standard deviations are reported above for continuous variables while N and percent of the sample are reported for categorical variables. * = a significant difference between males and females at *P* < 0.05. # = a significant difference between MZ and DZ twins at *P* < 0.05. MZ = monozygotic, DZ = dizygotic, IQ = intelligence quotient, GPA = grade point.
**Table S2.** Individual level, Cotwin, and MZ Cotwin Model Results for Trails A. The top table includes frequency of adolescent alcohol use as a predictor. The bottom table includes frequency of adolescent intoxication as a predictor. Bolded values represent estimates where the corresponding uncorrected *p*‐value is <0.05.
**Table S3.** Individual level, Cotwin, and MZ Cotwin Model Results for Trails B. The top table includes frequency of adolescent alcohol use as a predictor. The bottom table includes frequency of adolescent intoxication as a predictor. Bolded values represent estimates where the corresponding uncorrected *p*‐value is <0.05.
**Table S4.** Individual level, Cotwin, and MZ Cotwin Model Results for Classic Stroop. The top table includes frequency of adolescent alcohol use as a predictor. The bottom table includes frequency of adolescent intoxication as a predictor. Bolded values represent estimates where the corresponding uncorrected *p*‐value is <0.05.
**Table S5.** Individual level, Cotwin, and MZ Cotwin Model Results for California Stroop. The top table includes frequency of adolescent alcohol use as a predictor. The bottom table includes frequency of adolescent intoxication as a predictor. Bolded values represent estimates where the corresponding uncorrected *p*‐value is <0.05.
**Table S6.** Individual level, Cotwin, and MZ Cotwin Model Results for Vocabulary. The top table includes frequency of adolescent alcohol use as a predictor. The bottom table includes frequency of adolescent intoxication as a predictor. Bolded values represent estimates where the corresponding uncorrected *p*‐value is <0.05.
**Table S7.** Individual level, Cotwin, and MZ Cotwin Model Results for Block Design. The top table includes frequency of adolescent alcohol use as a predictor. The bottom table includes frequency of adolescent intoxication as a predictor. Bolded values represent estimates where the corresponding uncorrected *p*‐value is <0.05.
**Table S8.** Individual level, Cotwin, and MZ Cotwin Model Results for Digit Symbol. The top table includes frequency of adolescent alcohol use as a predictor. The bottom table includes frequency of adolescent intoxication as a predictor. Bolded values represent estimates where the corresponding uncorrected *p*‐value is <0.05.
**Table S9.** Individual level, Cotwin, and MZ Cotwin Model Results for Forward Digit Span. The top table includes frequency of adolescent alcohol use as a predictor. The bottom table includes frequency of adolescent intoxication as a predictor. Bolded values represent estimates where the corresponding uncorrected *p*‐value is <0.05.
**Table S10.** Individual level, Cotwin, and MZ Cotwin Model Results for Backward Digit Span. The top table includes frequency of adolescent alcohol use as a predictor. The bottom table includes frequency of adolescent intoxication as a predictor. Bolded values represent estimates where the corresponding uncorrected *p*‐value is <0.05.
**Table S11.** Individual level, Cotwin, and MZ Cotwin Model Results for Mental Rotation Test. The top table includes frequency of adolescent alcohol use as a predictor. The bottom table includes frequency of adolescent intoxication as a predictor. Bolded values represent estimates where the corresponding uncorrected *p*‐value is <0.05.
**Table S12.** Individual level, Cotwin, and MZ Cotwin Model Results for Object Location Memory. The top table includes frequency of adolescent alcohol use as a predictor. The bottom table includes frequency of adolescent intoxication as a predictor. Bolded values represent estimates where the corresponding uncorrected *p*‐value is <0.05.
**Table S13.** FDR corrected *p*‐values for associations between adolescent alcohol use and young adult cognitive outcomes. The adjusted *p*‐value column represents the false discovery rate (FDR) adjusted *p*‐value. This correction was made across cognitive tasks within each analytic level (correlation, individual level, co‐twin, MZ co‐twin).
**Table S14.** FDR corrected *p*‐values for associations between adolescent alcohol intoxication and young adult cognitive outcomes. The adjusted *p*‐value column represents the false discovery rate (FDR) adjusted *p*‐value. This correction was made across cognitive tasks within each analytic level (correlation, individual level, co‐twin, MZ co‐twin).
**Table S15.** Moderating effects of sex and adolescent alcohol on young adult cognition. Estimates (betas) reported above are the interaction term between sex and either frequency of adolescent alcohol use or frequency of adolescent alcohol intoxication. *P*‐values are uncorrected.

**Data S1.** Supplemental Methods

**Figure S1.** Comparison of Individual Level Models with and without Age 22 drinks per week included as a covariate.
**Figure S2.** Comparison of Individual Level Models.

## Data Availability

Research data are not shared owing to Finnish data privacy laws.
