# Supplementary material for: Association between adolescent alcohol use and cognitive function in young adulthood: A co‐twin comparison study
Source: Addiction. 2024 Aug 6;119(11):1947–55. doi: 10.1111/add.16629 (PMC11524136; doi:10.1111/add.16629)
Supplement: Supplementary file 2 — Data S1. Supplemental Methods [file ADD-119-1947-s001.docx]

Supplemental Methods

Neurocognitive Assessments

*Trail Making Test.* This task has two parts which both require participants to connect a series of 25 targets (Närhi et al., 1997; Tombaugh, 2004). For the first part, the targets are numbered 1 through 25 and the participant must connect them in chronological order. For the second part, half the targets are numbered 1 through 13 and the other half are lettered A through L. The participants must connect the targets in sequential order alternating between numbers and letters. Both tasks are timed with the goal being to correctly connect the targets as quickly as possible. A lower score, representing faster task completion, represents better cognitive ability with part A assessing processing speed and part B assessing set-shifting.

*California Stroop Test*. Participants were shown four lists: 1) 50 rectangular patches of different colors (red, blue, and green) arranged in a random order where participants should name the colors as quickly as possible, 2) 50 color words (RED, BLUE, and GREEN) printed in black ink and arranged in a random order where participants should read the word out loud as quickly as possible, 3) 50 color words (RED, BLUE, and GREEN) but the words are printed in a color ink that is different from the word where participants must name the color of the ink as quickly as possible, 4) the same list and rules as trial 3 except if the word is surrounded by a rectangle the participant must read the word printed not the color of the ink (Comalli et al., 1962; Homack & Riccio, 2004). Participants completed a practice of 10 words before each new trial type. Participants were timed when reciting these lists with lower times representing better cognitive ability.

*Wechsler Adult Intelligence Scale (WAIS).* Participants completed the Vocabulary and Block Design subtests from the WAIS-R and the Digit Symbol subtest from the WAIS III (Wechsler, 1997). For Vocabulary, participants must correctly define the words that are presented to them. In this version participants were presented every other word on the word list. The words presented progressively increased in difficulty. For Block Design, participants must rearrange blocks with white and red sides to make the pattern depicted on the page. Patterns are presented in increasing levels of difficultly. For Digit Symbol, participants are asked to transcribe a code by matching symbols to numbers as fast as possible in 90 seconds.

*Wechsler Memory Scale-R: Digit Span*. In the forward condition participants repeated the numbers in the same order as the they were presented by the experimenter. In the reverse condition, participants repeated the numbers in the reverse order as they were presented by the experimenter. For both conditions, length of a digit span increased by one digit after two trials of the same length (Wechsler, 1987).

*Mental Rotation Test.* Participants were asked to mentally rotate two-dimensional drawings of three-dimensional objects. In each trial, participants were asked to identify the two out of four targets that were correctly rotated (Peters et al., 1995). This task assesses mental rotation ability with a greater number of correct responses indicating better spatial processing.

*Object Location Memory.* Participants were shown drawings of 27 objects for one minute with the instructions to memorize the objects. After a minute, they were shown drawings of the same 27 objects but in 14 of the drawings the object had been moved. Participants had one minute to correctly identify whether the object was in a new location. This task assesses incidental memory. Data for this task are only available on a subset of the sample (N=339) due to the task being added to the neurocognitive battery midway through data collection.

References

Comalli, P. E., Wapner, S., & Werner, H. (1962). Interference Effects of Stroop Color-Word Test in Childhood, Adulthood, and Aging. *The Journal of Genetic Psychology*, *100*(1), 47–53. https://doi.org/10.1080/00221325.1962.10533572

Homack, S., & Riccio, C. A. (2004). A meta-analysis of the sensitivity and specificity of the Stroop Color and Word Test with children. *Archives of Clinical Neuropsychology: The Official Journal of the National Academy of Neuropsychologists*, *19*(6), 725–743. https://doi.org/10.1016/j.acn.2003.09.003

Närhi, V., Räsänen, P., Metsäpelto, R.-L., & Ahonen, T. (1997). Trail Making Test in Assessing Children with Reading Disabilities: A Test of Executive Functions or Content Information. *Perceptual and Motor Skills*, *84*(3_suppl), 1355–1362. https://doi.org/10.2466/pms.1997.84.3c.1355

Peters, M., Laeng, B., Latham, K., Jackson, M., Zaiyouna, R., & Richardson, C. (1995). A Redrawn Vandenberg and Kuse Mental Rotations Test—Different Versions and Factors That Affect Performance. *Brain and Cognition*, *28*(1), 39–58. https://doi.org/10.1006/brcg.1995.1032

Tombaugh, T. N. (2004). Trail Making Test A and B: Normative data stratified by age and education. *Archives of Clinical Neuropsychology*, *19*(2), 203–214. https://doi.org/10.1016/S0887-6177(03)00039-8

Wechsler, D. (1987). *The Wechsler Memory Scale, Revised (WMS-R) administration and scoring manual.* Psychological Corporation.

Wechsler, D. (1997). *Wechsler Adult Intelligence Scale: Administration and scoring manual*. Psychological Corporation.
