# Supplementary material for: Association between adolescent alcohol use and cognitive function in young adulthood: A co‐twin comparison study
Source: Addiction. 2024 Aug 6;119(11):1947–55. doi: 10.1111/add.16629 (PMC11524136; doi:10.1111/add.16629)
Supplement: Supplementary file 3 — Figure S1. Comparison of Individual Level Models with and without Age 22 drinks per week included as a covariate. Figure S2. Comparison of Individual Level Models. [file ADD-119-1947-s003.docx]

Supplemental Figures

Figure S1. Comparison of Individual Level Models with and without Age 22 drinks per week included as a covariate.


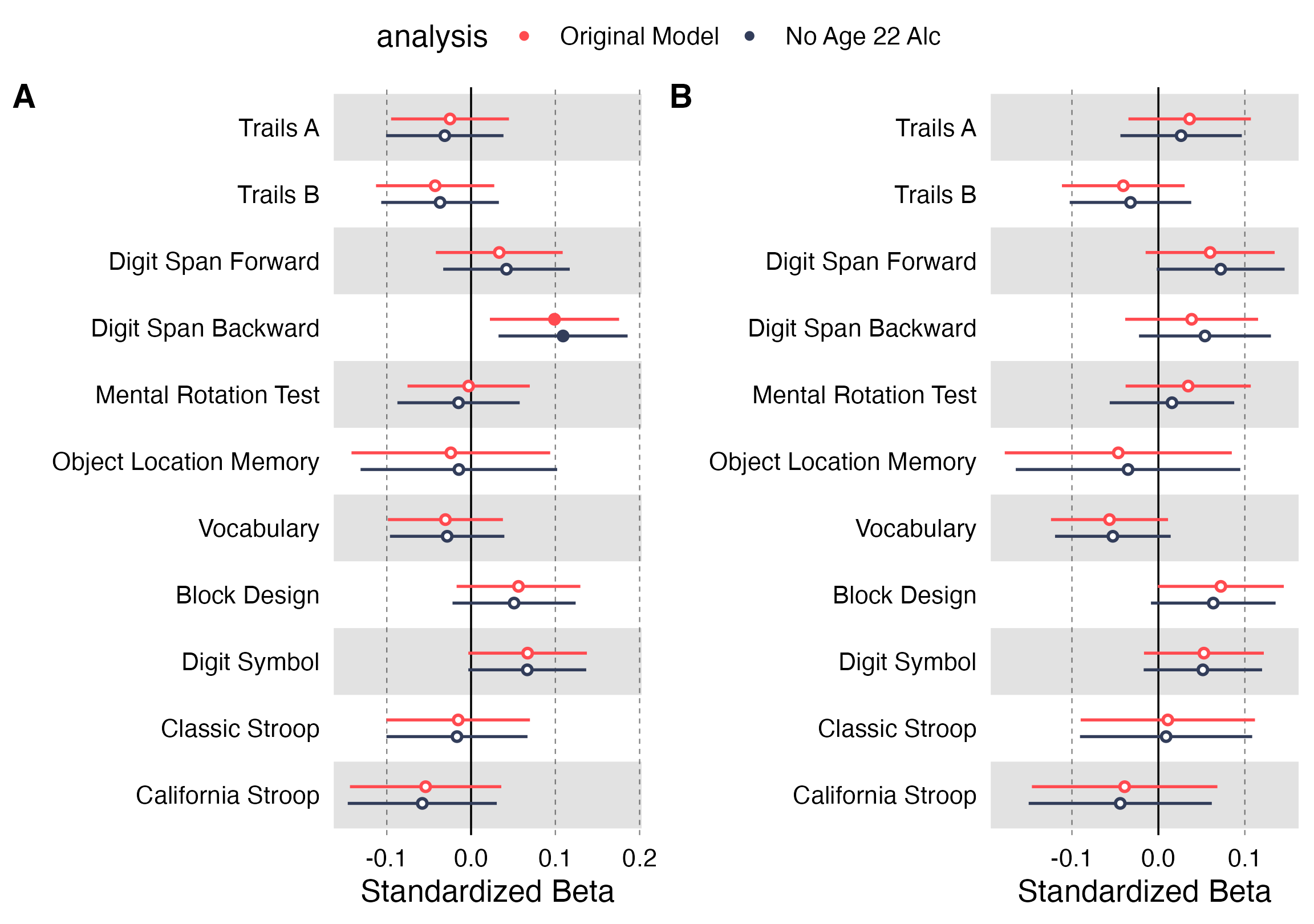


Note: Error bars represent 95% confidence intervals. Filled in circles represent p <0.05. Panel A is frequency of alcohol use as predictor. Panel B is frequency of intoxication as a predictor.

Figure S2. Comparison of Individual Level Models


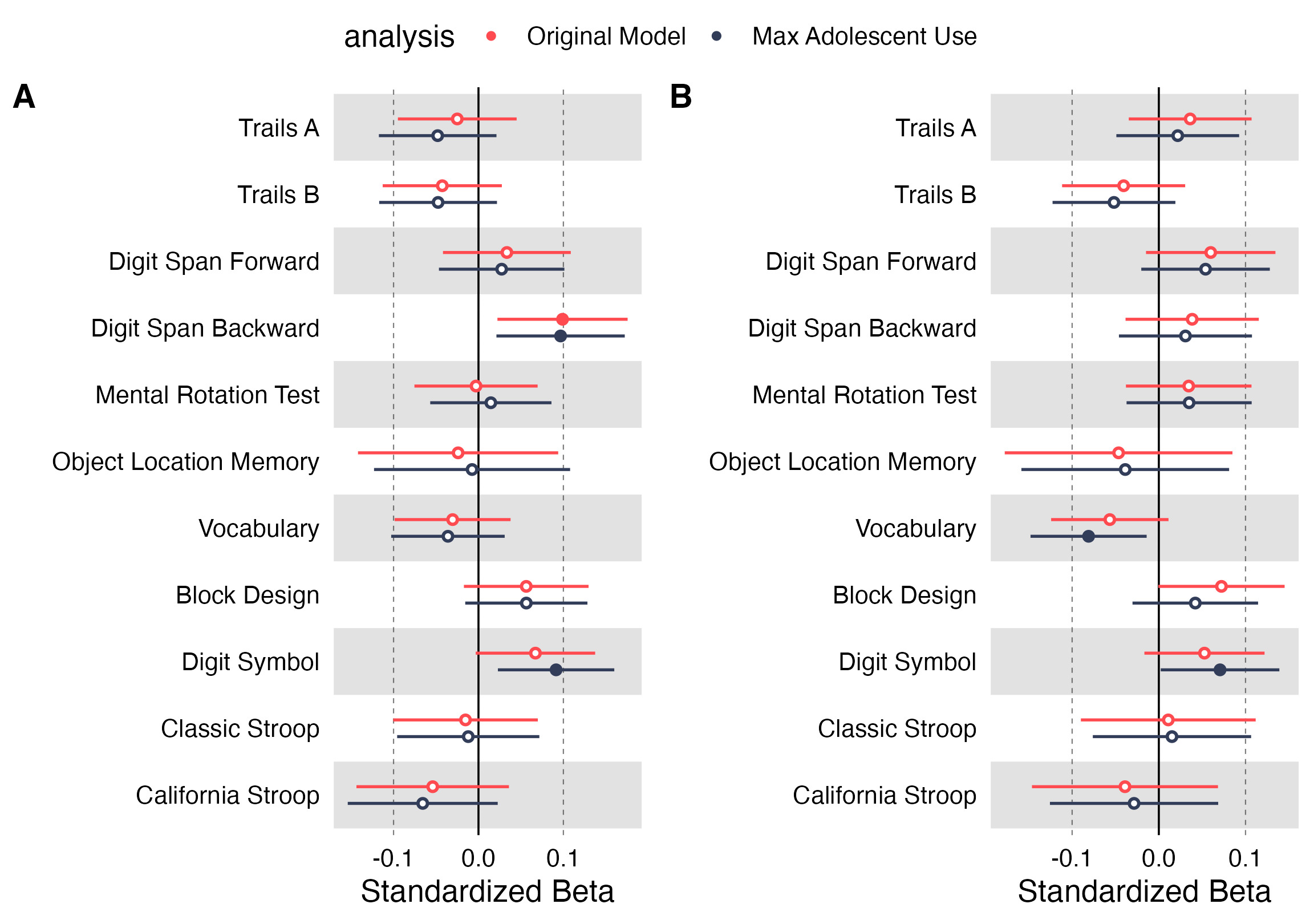


Note: Error bars represent 95% confidence intervals. Filled in circles represent p <0.05. Panel A is frequency of alcohol use as predictor. Panel B is frequency of intoxication as a predictor.
